# A novel pathogenic missense variant in *CNNM4* underlying Jalili syndrome: Insights from molecular dynamics simulations

**DOI:** 10.1002/mgg3.902

**Published:** 2019-07-25

**Authors:** Asia Parveen, Muhammad U. Mirza, Michiel Vanmeert, Javed Akhtar, Hina Bashir, Saadullah Khan, Saqib Shehzad, Matheus Froeyen, Wasim Ahmed, Muhammad Ansar, Naveed Wasif

**Affiliations:** ^1^ Institute of Molecular Biology and Biotechnology (IMBB), Centre for Research in Molecular Medicine (CRiMM) The University of Lahore Lahore Pakistan; ^2^ Faculty of Life Sciences University of Central Punjab (UCP) Lahore Pakistan; ^3^ Department of Pharmaceutical Sciences, REGA Institute for Medical Research, Medicinal Chemistry University of Leuven Leuven Belgium; ^4^ Department of Biochemistry Sharif Medical and Dental College Lahore Pakistan; ^5^ Department of Biotechnology and Genetic Engineering Kohat University of Science and Technology (KUST) Kohat Pakistan; ^6^ Department of Biochemistry, Faculty of Biological Sciences Quaid‐i‐Azam University Islamabad Pakistan; ^7^ Institute of Human Genetics University of Ulm & University Hospital Ulm Germany; ^8^ Institute of Human Genetics University Hospital Schleswig‐Holstein Kiel Germany

**Keywords:** CBS domain, *CNNM4*, Jalili Syndrome, MD Simulations, missense variant

## Abstract

**Background:**

Jalili syndrome (JS) is a rare cone‐rod dystrophy (CRD) associated with amelogenesis imperfecta (AI). The first clinical presentation of JS patients was published in 1988 by Jalili and Smith. Pathogenic mutations in the Cyclin and CBS Domain Divalent Metal Cation Transport Mediator 4 (CNNM4) magnesium transporter protein have been reported as the leading cause of this anomaly.

**Methods:**

In the present study, a clinical and genetic investigation was performed in a consanguineous family of Pakistani origin, showing characteristic features of JS. Sanger sequencing was successfully used to identify the causative variant in *CNNM4*. Molecular dynamics (MD) simulations were performed to study the effect of amino acid change over CNNM4 protein.

**Results:**

Sequence analysis of *CNNM4* revealed a novel missense variant (c.1220G>T, p.Arg407Leu) in exon‐1 encoding cystathionine‐β‐synthase (CBS) domain. To comprehend the mutational consequences in the structure, the mutant p.Arg407Leu was modeled together with a previously reported variant (c.1484C>T, p.Thr495Ile) in the same domain. Additionally, docking analysis deciphered the binding mode of the adenosine triphosphate (ATP) cofactor. Furthermore, 60ns MD simulations were carried out on wild type (p.Arg407/p.Thr495) and mutants (p.Arg407Leu/p.Thr495Ile) to understand the structural and energetic changes in protein structure and its dynamic behavior. An evident conformational shift of ATP in the binding site was observed in simulated mutants disrupting the native ATP‐binding mode.

**Conclusion:**

The novel identified variant in *CNNM4* is the first report from the Pakistani population. Overall, the study is valuable and may give a novel insight into metal transport in visual function and biomineralization.

## 1 INTRODUCTION

Cone‐rod dystrophy (CRD; MIM 120970) and amelogenesis imperfecta (AI; MIM 204700), collectively known as Jalili syndrome (JS, MIM 217080), were first reported by Jalili and Smith, (1988). JS is inherited in an autosomal recessive fashion. CRDs are a clinically and genetically heterogeneous group of progressive retinal disorders. Clinically, CRD manifests as an initial loss of central vision, reduced color perception, and photophobia. While the disease progresses consequent night blindness and restricted visual field surface (Moore, 1992). The age of onset in CRDs is variable, but most patients are diagnosed in the first two decades of their lives. Representation of CRD can be syndromic; however, in most cases, it is reported as an isolated defect. AI is an inherited abnormality of tooth enamel. The thin enamel results from hypomaturation and hypoplasticity, poor mineralization, or a combination of both. Isolated forms of CRD and AI can be inherited in an autosomal dominant, autosomal recessive, or X‐linked pattern (Aldred et al., 1992; Witkop, 1988). CRD and AI can be linked with an extra‐systemic clinical picture involving various tissues and organs.

The Cyclin and CBS Domain Divalent Metal Cation Transport Mediator (CNNM) family proteins possess a highly conserved multi‐domain structure including two tandem CBS domains. Several studies have reported the physiological importance of the CBS domain and its essential role in the transport of Mg^2+^ (Chen, Yang, Yang, Fakih, Kozlov, & Gehring, 2018; Funato & Miki, 2018; Giménez‐Mascarell et al., 2019; Hattori et al., 2009; Ignoul & Eggermont, 2005). Several biochemical studies on CBS‐containing proteins like inosine‐5’‐monophosphate dehydrogenase (IMPDH), AMP‐activated protein kinase (AMPK), inosine‐5’‐monophosphate dehydrogenase (IMPDH), and chloride channel (ClC) family proteins, have explained the importance of interactions of adenine nucleotides to CBS domain (Baykov, Tuominen, Tuominen, & Lahti, 2011; Scott et al., 2004). The amino acid substitutions in CBS are linked to the loss of activity, which highlights the vital role of adenosine nucleotides (Scott et al., 2004). Likewise, in CNNM family proteins, CBS domains play a functionally important role in Mg^2+^ efflux, probably through interactions with adenosine triphosphate (ATP) (Hirata, Funato, Funato, Takano, & Miki, 2014).

Mutations in other CBS domain‐containing proteins have also been associated with several hereditary diseases, including: myotonia congenita (MIM 160800), homocystinuria (MIM 236200), osteopetrosis (MIM 259700), Bartter syndrome (MIM 241200), retinitis pigmentosa (MIM 180105), idiopathic generalized epilepsy (MIM 600669), familial hypertrophic cardiomyopathy with Wolff‐Parkinson‐White syndrome (MIM 600858), and Dent's disease (MIM 300009) (Ignoul & Eggermont, 2005).

Cyclin and CBS Domain Divalent Metal Cation Transport Mediator 4 (CNNM4)is presumed to be involved in the transport of magnesium ions, which are essential for homeostasis in humans (Meyer et al., 2010). The expression of CNNM4 has been confirmed in the neural retina and ameloblasts of the developing teeth (Parry et al., 2009; Polok et al., 2009). It has been documented that CNNM4 plays a crucial role in the mineralization process during the formation of hydroxyapatite (Parry et al., 2009; Polok et al., 2009). Mineral deficiency has been observed in the dental enamel of AI patients due to the disturbances in Mg^2+^ transport (Polok et al., 2009).

Here, we report the identification of a novel homozygous missense variant (c.1220G>T, p.Arg407Leu) in *CNNM4* in a consanguineous Pakistani family showing prominent features of JS. As the proposed variant was present in the significantly important ATP‐binding site of the CBS domain, therefore, a molecular modeling approach was designed to investigate the dynamic consequences of ATP binding upon alteration. We further analyzed the structural impact of this novel amino acid substitution (p.Arg407Leu) through MD simulations of an already reported variant (c.1484C>T, p.Thr495Ile) which is stated in CRD patients (Abu‐Safieh et al., 2012).

## 2 MATERIALS AND METHODS

### 2.1 Ethics approval, subjects enrollment, and genomic DNA extraction

The principles of the Declaration of Helsinki were strictly followed to accomplish this study. Research protocols and working on human samples were approved by the Institutional Review Board (IRB) (VC‐UOL/240712/A01‐UOL) of The University of Lahore, Lahore, Pakistan. Informed consent, followed by a signature or a thumb impression, was obtained from all the patients and the healthy volunteers involved in the study. Venous blood samples were collected from available affected (IV‐3, IV‐4, and IV‐5) and unaffected (III‐2, IV‐1, and IV‐2) individuals (Figure S1). Genomic DNA was extracted following a standard protocol (Miller, Dykes, Dykes, & Polesky, 1988). Patient IV‐3 was subjected to ophthalmoscopy followed by an electroretinography (ERG) to conclude the visionary defects and an oral examination followed by an orthopantomogram (OPG) to understand the dental conditions (Figure S2).

### 2.2 Mutation screening of *CNNM4*

Identification of the mutation in *CNNM4* and further cosegregation analysis was carried out by Sanger sequencing. Sanger sequencing and mutational analysis were performed as described by (Jelani, Wasif, Wasif, Ali, Chishti, & Ahmad, 2008). Genomic sequences of *CNNM4* (NM_020184.4) were downloaded from the University of California Santa Cruz (UCSC) genome database browser (http://genome.ucsc.edu/cgi-bin/hgGateway). AmplifX v1.5.4 software was used for designing the primers (http://crn2m.univ-mrs.fr/pub/amplifx). Homologene (http://www.ncbi.nlm.nih.gov/homologene/) was consulted to examine the evolutionary conservation in CNNM4 orthologs. The functional context of the identified variant was predicted through PolyPhen 2.0 (Adzhubei, Jordan, & Sunyaev, 2013) and I‐Mutant 3.0 (Capriotti, Fariselli, Fariselli, Rossi, & Casadio, 2008). PolyPhen 2.0 is based on a combination of sequence/structure attributes and uses a naïve Bayesian classifier to describe amino acid substitution. The predicted output levels of “probably” and “possibly damaging” are classified as functionally significant and correspond to the value ≤0.5 while “benign” is classified as tolerated (≥0.51). I‐Mutant 3.0 is a support vector machine (SVM)‐based tool which estimates Gibbs free energy change (ΔΔG) upon mutation into three classes: neutral variation (−0.5 ≤ ΔΔG ≥ 0.5 kcal/mol), the significant decrease of stability (<−0.5 kcal/mol), and a substantial increase of stability (>0.5 kcal/mol), while ΔΔG value represents the stability of protein upon mutation(s).

### 2.3 Preparation of starting structure

The X‐ray crystal structure of human‐CNNM4 was obtained from the Protein Data Bank (PDB ID: 4IY3). For the current study, two variants (a novel, c.1220G>T, p.Arg407Leu and an already reported, c.1484C>T, p.Thr495Ile) were selected because they were present within‐5Å of the center of the ATP‐binding site. The Swiss‐model program was applied to generate the 3D coordinates of *mut*‐Arg407Leu and *mut*‐Thr495Ile by substituting specific residues using the human‐CNNM4 crystal structure as a template. The quality of each predicted model was checked using PROCHECK (Laskowski, MacArthur, MacArthur, Moss, & Thornton, 1993), ERRAT (Colovos & Yeates, 1993), and ProSA‐Web (Wiederstein & Sippl, 2007). Due to the unavailability of an ATP cocrystallized structure for CNNM4, mouse‐CNNM2 complexed with ATP‐Mg was retrieved from the PDB (PDB ID: 4P1O) to get the native conformation of ATP ligand. The structural analysis of both proteins showed a root mean square deviation (RMSD) of 1.063Å when superimposed (Figure S3). In the present study, mouse CNNM2‐ATP cocrystalized structure was used as a control to understand the dynamic behavior of ATP upon reported mutations. ATP structure was extracted from mouse‐CNNM2 complex, and hydrogen atoms and gasteiger charges were added using chimera 10.2. Finally, the ATP molecule was docked with *wt‐*CNNM4, *mut*‐Arg407Leu, and *mut*‐Thr495Ile using AutoDock Vina. Details of protein preparation, optimization, and minimization for molecular docking were described elsewhere (Saeed et al., 2018). The complexes were analyzed using Chimera 1.13 (Pettersen et al., 2004).

### 2.4 Molecular dynamics simulations

Molecular dynamics (MD) simulations of 60ns were performed to check the backbone stability of top‐ranked and readily available ligands using the AMBER 16.0 software package (Case et al., 2014). All calculations were performed on a Linux‐based operating system. Counterions and solvent were defined using the Amber ff14SB force field (Maier et al., 2015). The build‐in antechamber method was used with the AMBER force field (GAFF) to generate force field parameters for all selected compounds. The terminal leap (tleap) module of AMBER was used to produce all essential parameters by considering residues at their default protonation states at neutral pH value. Prior to energy minimization, all complexes were neutralized by adding Na^+^ ions around the complex at different locations and centered in a dodecahedral simulation box with a 12Å distance from the edge of the box and solvated using TIP3P water molecules. Particle mesh Ewald method with a nonbounded cut‐off of 10Å was used to set the periodic boundary conditions. Integration steps of 2fs were established, and the SHAKE algorithm was used to constrain X‐H bond lengths involving hydrogen bonds. The solvated and neutralized system was subjected to energetic minimization using 5,000 steps of steepest descent method followed by the conjugate gradient method. Afterward, the system was gradually heated from 0 to 300 K with 50ps and maintained at 300K with restraints on the complex. Solvent density was reached after 500ps after which the system was equilibrated for another 500ps. Finally, a 20ns relaxed MD calculation was performed under isobaric and isothermal conditions (*p* = 1.0 atm, T = 300K). Coordinate trajectories were collected after every 2ps for the whole production run, and CPPTRAJ modules of AMBER 16 were implied for trajectories analyses (Roe & Cheatham, 2013). The RMSD was computed using the CPPTRAJ modules in AmberTools, indicating protein conformational stability.

## 3 RESULTS

### 3.1 Clinical features

We report the case of a consanguineous Pakistani family (Figure S1) with clinical features of JS. The pedigree consists of three affected (IV‐3, IV‐4, IV‐5) and two unaffected (IV‐1, IV‐2) individuals born to an unaffected couple (III‐1, III‐2), suggesting autosomal recessive inheritance. Clinical investigation of unaffected grandparents (both paternal and maternal) did not reveal any disease symptoms. All JS patients were born after uncomplicated full‐term pregnancies. Age of onset of CRD ranged from 5 to 8 years after birth in affected individuals, based on information collected from family elders at the time of visit. Studied subjects were 9, 11, and 13 years of age at the time of their visit to University Hospital, The University of Lahore. All affected members showed photophobia, amblyopia, atrophic macular degeneration, gradual progression of the optic disc, and fine pendular nystagmus (Figure S2a,b). The light‐adapted ERG results showed the cone dystrophy while the dark‐adapted ERG testified the severely impaired rod response (Figure S2c). Teeth of affected individuals showed clinical signs of hypomaturative/hypoplastic AI with yellow to brown discoloration (Figure S2d,e). The OPG results of an affected member (IV‐3) also coincided with the clinical pictures, showing grossly carious teeth. The maxilla contained a total of 10 teeth, which are malformed, discolored, and periodontally involved. The mandibular arch had four teeth in similar conditions. The mandibular ridge was resorbed due to the loss of teeth (Figure S2f). No pathological findings, cyst or tumor, were apparent in the mandible or maxilla.

### 3.2 Mutation analysis

**Figure 1:**
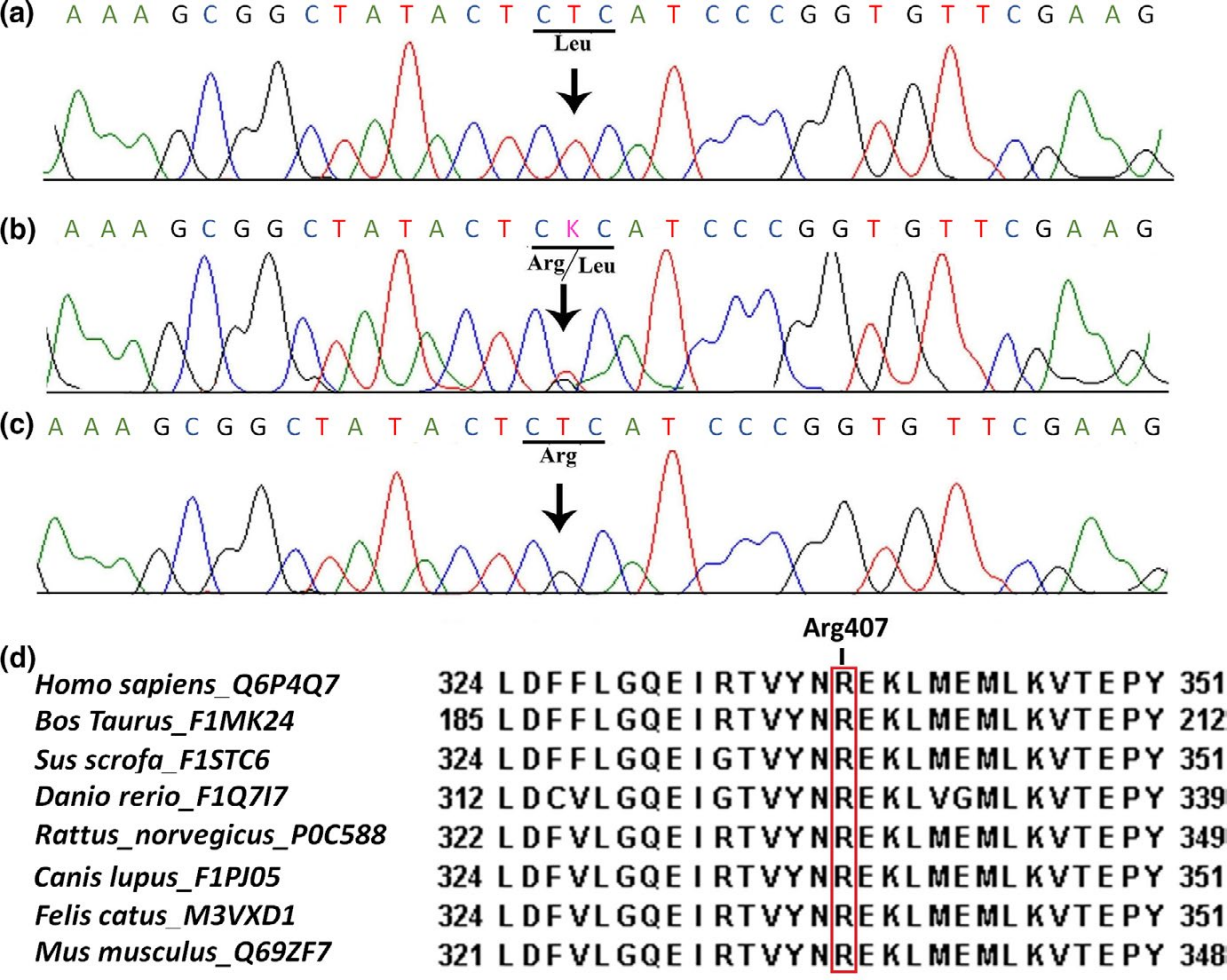
Sequence analysis of a novel variant (c.1220G>T, p.Arg407Leu) identified in Cyclin and CBS Domain Divalent Metal Cation Transport Mediator 4; (a) represents an affected individual (IV‐3) with the homozygous sequence variation (CGC‐Arg_CTC‐Leu) (b) the heterozygous carrier (III‐2) and (c) represents the wildtype nucleotide sequence in an unaffected individual (IV‐1). Panel (d) shows partial amino acid sequence comparison of human Cyclin and CBS Domain Divalent Metal Cation Transport Mediator 4 protein with other orthologs. A red box indicates arginine (R) 407 residue conserved across different species

Sequence analysis of *CNNM4* detected a homozygous G to T transversion at nucleotide position 1,220 (c.1220G>T) in affected individuals of the family (Figure 1a). This sequence change resulted in the substitution of arginine (CGC) to leucine (CTC) (Figure 1a‐c) at amino acid position 407 (p.Arg407Leu) in CNNM4. This novel missense variant (c.1220G>T, p.Arg407Leu) lies in exon‐1 which composes the CBS1 domain of the CNNM4. The identified variant is highly conserved across species (Figure 1d). ClinVar (https://submit.ncbi.nlm.nih.gov/clinvar/) has reserved an accession ID (SCV000902267) for this variant.

The rare variant has not yet been described in the Genome Aggregation Database (gnomAD, http://gnomad.broadinstitute.org/). PolyPhen 2.0 has classified this pathogenic variant (p.Arg407Leu) as probably damaging (Score 1.000), while I‐Mutant 3.0 has classified it as a disease‐causing variant.

### 3.3 Structural insights into the binding of ATP upon mutations

After classifying the clinical effect of the novel variant (c.1220G>T, p.Arg407Leu) as disease causing, the structural results were analyzed to investigate the changes in ATP binding over time. A previously reported pathogenic variant (c.1484C>T, p.Thr495Ile) was also analyzed for comparison. The biomolecular models were generated by docking ATP with the CNNM4 homology model for this analysis. The docking of ATP with the wild‐type variant of CNNM4 resulted in a complex with a binding affinity of −8.9 kcal/mol. The docking, with p.Arg407Leu, and p.Thr495Ile variants of CNNM4 resulted in interaction energy of −5.9 and 5.3 kcal/mol, respectively. This initial difference showed the less favorable energetic interaction upon mutations. The overall stereochemistry and structural quality of designed models were assessed using PROCHECK, ERRAT, and ProSA‐Web, of which a summary of obtained results is tabulated in Table 1.

**Table 1:** Evaluation of homology models by using PROCHECK, ERRAT, and ProSA‐Web

CNNM4	PROCHECK					ERRAT	ProSA
	Ramachandran plot statistics (%)				G‐factor		
	Core	Allowed	General	Disallowed	Overall score		
p.Arg407Leu	95	4.2	0	0.8	0.15	96.85	−6.21
p.Thr495Ile	94.2	5.3	0	0.8	0.11	92.9	−6.02

Note

Ramachandran plot qualities indicates the percentage of the residues present in the core, allowed, generally allowed, and disallowed regions of the plot: G‐factor represents Goodness factor which show the quality of covalent and overall bond/angle distances (above ‐ 0.5 indicates a reliable model): ERRAT and ProSA score show the calculated overall homology model quality score.

Abbreviations: CNNM4, Cyclin and CBS Domain Divalent Metal Cation Transport Mediator 4.

**Figure 2:**
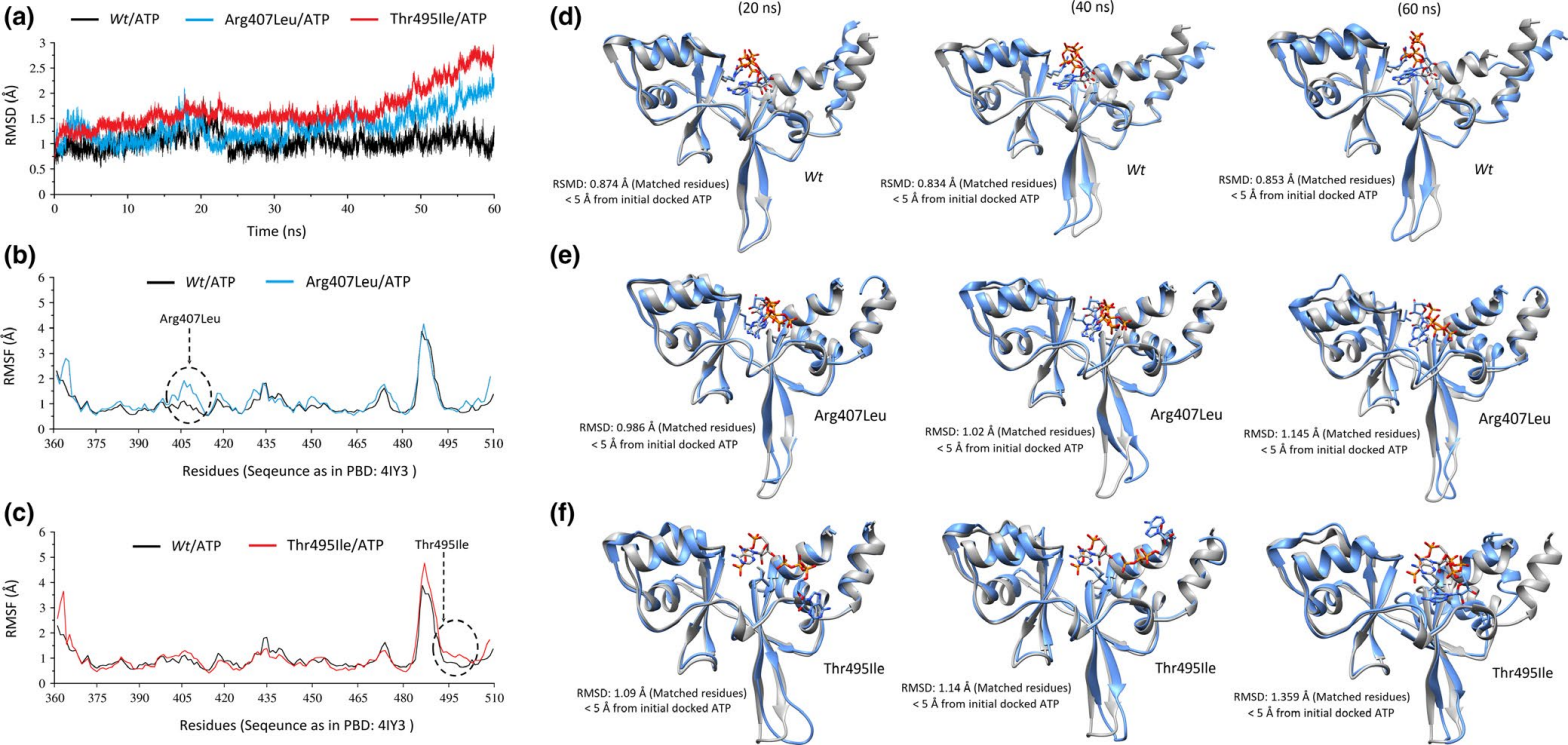
. The RMSD trajectories of the Cyclin and CBS Domain Divalent Metal Cation Transport Mediator 4 backbone atoms (a) throughout 60ns for wt (black), Arg407Leu (turquoise), and Thr495Ile (red); and RMSF of backbone atoms relative to wt are displayed for Arg407Leu (b), and Thr495Ile (c) respectively. The comparison of conformational changes after every 20ns concerning the starting structure is interactively presented (in the ribbon) for wt (d), Arg407Leu (e) and Thr495Ile (f), complexed with ATP molecule (in the sticks), respectively

To investigate structural changes upon alterations (p.Arg407Leu and p.Thr495Ile) in the ATP‐binding domain of CNNM4, the modeled structures (wild type and mutants) were subjected to 60ns MD simulations resulting in the elucidation of moderate to severe binding mode distortion with CNNM4 CBS domain. Structural analysis, in terms of stability, of these missense variants in comparison to its wild‐type (*wt)* variants, was carried out by RMSD and root mean square fluctuation (RMSF) calculations, shown in Figure 2a‐c. Conformational changes in mutant complexes compared with *wt*CNNM4/ATP are displayed with a 20ns interval in Figure 2d‐f.

**Figure 3:**
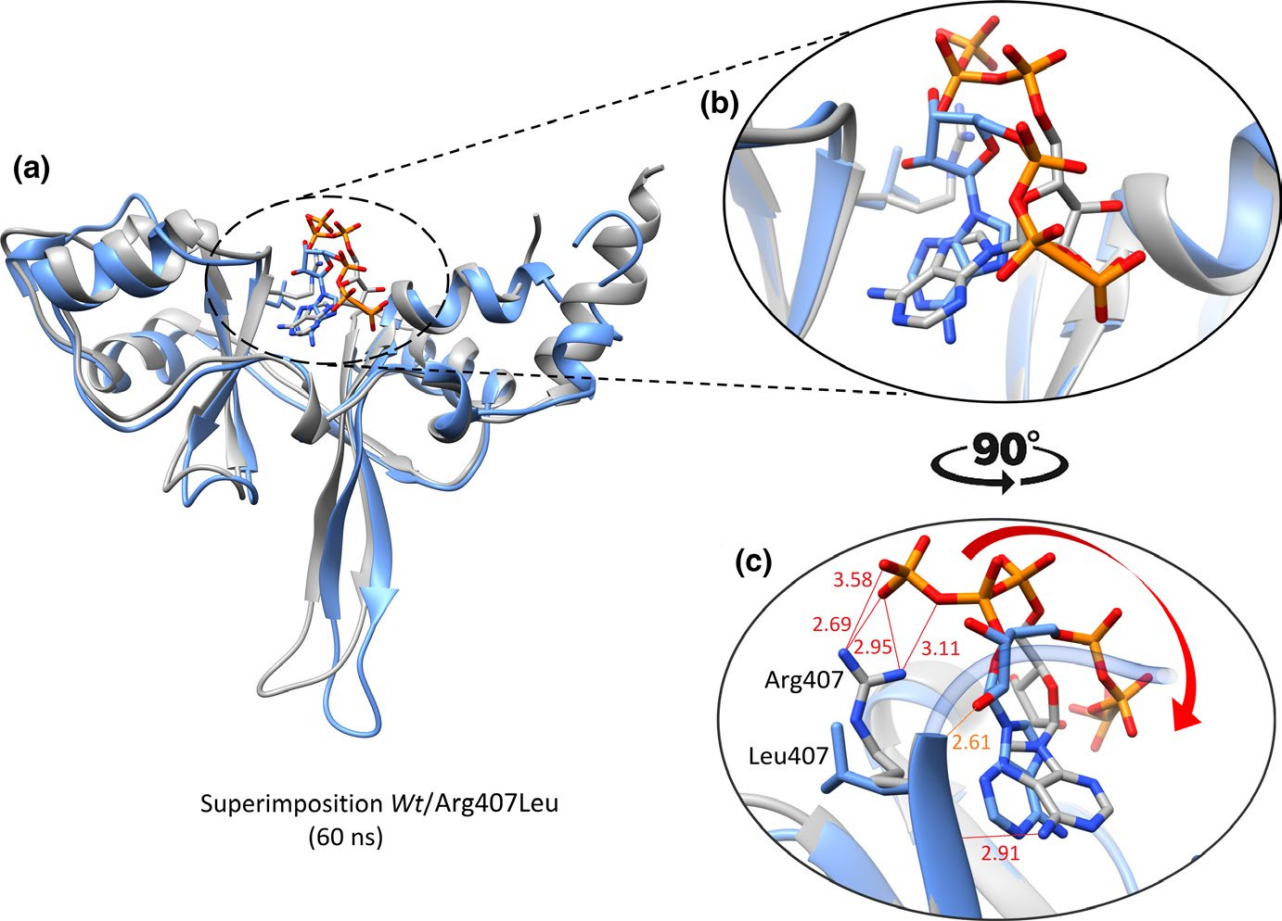
(a) The structural comparison of wt (in gray) and Arg407Leu (in blue) complexed with ATP molecule (with the same color code) after 60ns molecular dynamics simulation period. The formation of hydrogen bonds with distance in Angstrom (Å) between wt and Arg407Leu complexes are shown in close‐ups (b and c) while the movement of ATPtriphosphate is indicated with an arrow (in red)

The increasing all‐atoms backbone RMSD values for p.Arg407Leu (turquoise) and p.Thr495Ile (red) compared to the converging values for *wt* (black) indicates a destabilizing effect. The backbone atoms of *wt*CNNM4 display minor fluctuation after 10ns but the complex remained stable for the last ~ 35ns. Binding site residues, defined as present within 5Å of the docked ATP cofactor, fluctuated slightly (<1Å) with the cofactor remaining inside the binding pocket throughout the simulation period. In both mutants, the RMSD increases gradually in time with a maximum deviation of ~2.5Å in the last 10ns without further convergence (Figure 2a). Consistent with RMSD analysis, the RMSF of p.Arg407Leu (turquoise) shows notably increased fluctuation up to 1.2Å compared to wtCNNM4 (red) complex in the range of residues between 400 and 410, including the point mutation of interest (Figure 2b). Rotational and translational rearrangement of the ATP cofactor in the docked complex, with loss of essential interactions established by Arg407 and Thr495 in *wt*CNNM4, is evident from the obtained static conformations after 20, 40, and 60ns (Figure 2d). The translational movement of phosphates due to the presence of a hydrophobic leucine at position 407 instead of a hydrogen bond accepting arginine causes a slight lateral opening effect between the two top alpha‐helices flanking the ATP‐binding site which can be seen in conformations obtained after every 20ns (Figure 2e). The increased flexibility of p.Arg407Leu, causing displacement of ATP phosphates in the active site, was further comprehended by hydrogen bond analysis showing disruption throughout 60ns (Figure 3a‐c). Hydrogen bond traces were also analyzed for the simulated trajectory (Figure S4). For Arg407Leu, a stable and robust hydrogen bond with Arg407 is shifted towards a stable but weaker hydrogen bond interaction with Thr406. For Thr495Ile, a diluted network of hydrogen bonds became evident with a highly fluctuating on‐off hydrogen bond formation between the phosphate group of ATP and Arg407. Therefore, change of threonine to isoleucine at position 495 destabilizes the ATP interaction resulting in ATP migration away from the active site.

Hydrogen bond formation is by far the most critical factor which affects the wild‐type protein function upon nonsynonymous SNPs (Chen et al., 2001; Hunt et al., 2008; Zhang, Teng, Teng, Wang, Schwartz, & Alexov, 2010). Analysis of hydrogen bonds in conformations obtained after 60ns for both *wt*CNNM4 and p.Arg407Leu complexes indicate a substantial contribution of p.Arg407Leu which establishes four hydrogen bonds of ~<3.5Å with oxygen atoms of gamma (γ) phosphate of the ATP cofactor (Figure 3c) consistent with the formation of an ionic bridge between Arg407 and the terminal pyrophosphate to position the ATP cofactor. The rotational movement of the triphosphate moiety as a result of the variant p.Arg407Leu is highlighted in Figure 3.

**Figure 4:**
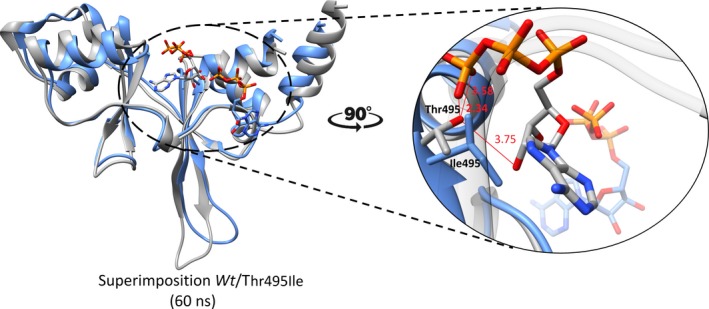
The structural comparison of wt (in gray) and Thr495Ile (in blue) complexed with ATP molecule (with the same color code) after 60ns molecular dynamics simulation period. The formation of hydrogen bonds with distance in Angstrom (Å) between wt and Thr495Ile complexes is shown in the right close‐up

The same insight was obtained after the MD simulation of p.Thr495Ile for which the RMSD and RMSF plots are shown in Figure 2a and 2c, respectively. The increasing average all‐atoms backbone RMSD for p.Thr495Ile (red) compared to the value for *wt*CNNM4 (black), together with increased regional flexibility around residues 490–500, as shown in the RMSF plot (Figure 2c), indicates a less stable complex formation with ATP. Compared to *wt*CNNM4, a severe distortion effect is seen with one of the alpha helices, flanking the ATP‐binding region, causing a rotational shift of ATP away from the binding site after a 60ns MD simulation (Figure 2f). The terminal pyrophosphate of ATP forms two alternating hydrogen bonds with Thr495 stabilizing the cofactor towards ionic bridge formation with Arg407. Mutation towards a hydrophobically interacting Ile495 disrupts this interaction and increases the conformational space, resulting in the release of ATP from the binding region, signifying the importance of Thr495 in cofactor stabilization (Figure 4). To further estimate the vibrational entropy energy (ΔΔSVib) and thermal stability (ΔΔG) upon these mutations, ENCoM server was utilized (Frappier, Chartier, Chartier, & Najmanovich, 2015), which indicated increased flexibility in p.Arg407Leu (ΔΔS_Vib_ ENCoM: 0.432 kcal.mol^−1^ K^−1^; ΔΔG: −0.822 kcal/mol as the destabilizing effect upon variation) and p.Thr495Ile (ΔΔS_Vib_ ENCoM: 0.583 kcal.mol^−1^ K^−1^; ΔΔG: −1.257 kcal/mol as the destabilizing effect upon variation). These thermodynamics predictions are consistent with the distortion effect in the ATP‐binding domain observed after MD simulations (Figure 2e,f).

Molecular mechanics/Generalized Born surface area (MM/GBSA) free binding energy of the complexes could not be calculated since the ATP was released from the binding site in case of Thr495Ile resulting in positive mm/gbsa values, incomparable with mm/gbsa values for the complexed ATP with wtCNNM4 CBS domain.

However, their overall biochemical mechanism of action and structural elucidation of the CBS domains of CNNM4 is presently unknown (Gómez García, Oyenarte, Oyenarte, & Martínez‐Cruz, 2011). With a molecular modeling approach, it is plausible to describe that CBS domains of CNNM4 bind directly to ATP, depending on the presence of Mg^2+^. The interactions with ATP may account for immediate Mg^2+^ transport (Chen, Kozlov, et al., 2018; Hirata et al., 2014). Given the small size of the metal ions, the strict positioning by Arg407 and Thr495, of the pyrophosphate chain, is hypothesized to be crucial. Therefore, the loss of interaction with both amino acids and the subsequent distortion of the ATP‐binding mode in the active site, together with the overall resulting complex instability, is hypothesized to be the cause of CNNM4 malfunction.

## 4 DISCUSSION

In the current study, we examined a Pakistani family exhibiting clinical symptoms consistent with JS. Sanger sequencing revealed a novel homozygous missense variant (c.1220G>T, p.Arg407Leu) in exon‐1 of *CNNM4*. The CNNM4 protein is a member of the CNNM family of Mg^2+^ transporters. CNNM4 (Q6P4Q7), has a DUF21 domain (residues 184–358) comprising four transmembrane helices (residues 182–204, 239–261, 265–287, and 294–316) and a leucine‐zipper (residues 188–209). Further composition of this protein contains one cyclic nucleotide monophosphate (cNMP)‐binding domain (residues 575–695), one cyclin‐box motif domain similar to the one present in ion channels and cNMP‐dependent kinases from residues 548–578, and two CBS domains (residues 377–438 and 445–511), which are believed to have a regulatory role in its biological activity (Gómez García et al., 2011).

JS is caused by mutations in *CNNM4* (Cyclin M4; MIM 607805), which encodes a protein, involved in the transport of metal ions, most likely magnesium (Mg) (Luder, Gerth‐Kahlert, Gerth‐Kahlert, Ostertag‐Benzinger, & Schorderet, 2013; Meyer et al., 2010), which is essential for proper function of the photoreceptors in the retina. CNNM4 is expressed both in the retina and the developing teeth (Luder et al., 2013; Parry et al., 2009). *CNNM4* mutations have revealed clinical consequences which are limited to retinal function in CRD and bio‐mineralization of teeth in AI (Parry et al., 2009). Twenty‐four *CNNM4* mutations, including a single base insertion, base pair duplication, missense changes, large deletions, and termination mutations have been characterized in patients suffering from JS around the world (Abu‐Safieh et al., 2013; Coppieters et al., 2014; Doucette et al., 2013; Huang et al., 2012; Jaouad et al., 2017; Kiessling, Mitter, Mitter, Langmann, & Müller, 2016; Lopez Torres, Schorderet, Schorderet, Valmaggia, & Todorova, 2015; Luder et al., 2013; Maia et al., 2018; Polok et al., 2009; Prasad et al., 2016; Rahimi‐Aliabadi et al., 2016; Topçu et al., 2017; Wang et al., 2015; Wawrocka et al., 2017; Zobor et al., 2012). These mutations presumably influence the divalent metal transporter function of CNNM4 protein. Mutants of CNNM4 in the complete absence of CBS domain have been identified to be incapable of promoting Mg^2+^ efflux. CNNM family proteins are evolutionary conserved Mg^+2^ transporters, and CBS domains perform a vital role in Mg^+2^ efflux (Wang et al., 2015).

Both missense variants (c.1220G>T, p.Arg407Leu; c.1484C>T, p.Thr495Ile) under study, are located in the conserved CBS region, lining the ATP‐binding pocket and therefore predicted to be pathogenic by Polyphen 2.0 and I‐mutant servers. Notably, both residues, being positioned inside the ATP‐binding pocket, are essential for the interaction with the ATP molecule by stabilizing its position. We have analyzed the structural and dynamic consequences of these variants in silico. The binding stability of the natural ATP cofactor in the active site of the target and overall dynamic behavior of the protein was studied in an explicit solvent environment using MD simulations comparing both pathogenic variants (p.Arg407Leu and p.Thr495Ile) with *wt*CNNM4. The molecular docking of ATP was performed with all CNNM4 models. Subsequently, the binding modes of ATP were investigated by subjecting the complexes to 60ns MD simulations to better comprehend the conformational stability and structural anomalies between wild type (p.Arg407/p.Thr495) and mutants (p.Arg407Leu/p.Thr495Ile).

The substitution of Arg407 with a smaller leucine residue shifted the mode of interaction from ionic bridge formation to hydrophobically interacting, additionally altering the orientation of flanking alpha helices, eventually distorting the ATP conformation within the binding pocket. This dynamic behavior and loss of crucial ionic interaction projected the noticeable movement of ATP triphosphate moiety over time (Figures 2e and 3).

The second pathogenic substitution of Thr495 by Ile495 resulted in an overall distorted conformation with the release of the ATP ligand from the binding pocket after 60ns simulation (Figure 4). In *wt*CNNM4, both Arg407 and Thr495 showed their pivotal role in maintaining the favorable conformation of ATP, thereby stabilizing the CBS domain.

The binding of ATP is likely to be involved in Mg^2+^ efflux, and mutations in the CBS domain abrogated the interaction with ATP (de Baaij et al., 2012; Hirata et al., 2014; Scott et al., 2004). These results correlate with the reported loss‐of‐function mutation in CNNM2 (c.1702C>T, p.Thr568Ile) located inside the ATP‐binding pocket (Stuiver et al., 2011).

The extensive MD simulations of p.Arg407Leu, and p.Thr495Ile variants provided crucial insights into the binding dynamics of cofactor ATP in the Mg^2+^ transporter CNNM4. These missense variants distort the protein structure and prevent the stabilizing nature of Arg407 and Thr495 towards the ATP cofactor. Furthermore, this dynamic consequences upon mutation was evident from the increased vibrational entropy energy between wild type and mutants (p.Arg407Leu/p.Thr495Ile) which escalated the flexibility of ATP‐binding domain, and the underlying vibrational entropy contributed significantly to the binding free energies of proteins (Frappier et al., 2015; Goethe, Fita, Fita, & Rubi, 2014). Delocalization of the triphosphate moiety in p.Arg407Leu mutant and complete release of ATP from the binding region in p.Thr495Ile are hypothesized to prevent the usual catalysis of Mg^2+^ transporters.

Hence, the comprehensive MD can better estimate the dynamic behavior of proteins after mutations, additionally providing molecular insights of interactions involved in protein catalytic function (Adcock & McCammon, 2006; Karplus & Kuriyan, 2005; Karplus & McCammon, 2002). The presented in silico analysis revealed that the conformational stability, because of CNNM4 variants, is considerably reduced as compared to its wild type, ultimately leading to a lack of ATP‐binding capability of CNNM4.

## CONFLICT OF INTERESTS

The authors have no potential conflict of interest in the form of relationship, and finances which could have affected the current study.

## AUTHORS’ CONTRIBUTIONS

AP, JA, HB, MA, and SK enrolled the patients and made the clinical diagnoses and wrote the clinical synopsis. JA and AP performed Sanger sequencing and verified the variant. MUM, MV, and SS did MD simulations studies. MF provided the consultation over the major MD simulations revisions and revised the structural analysis. NW and AP wrote the early draft of this manuscript and revised it. WA critically reviewed and finalized the manuscript. All authors agree upon the final draft and are responsible for the content of the manuscript.
