# Supplementary figures and images for: A novel pathogenic missense variant in CNNM4 underlying Jalili syndrome: Insights from molecular dynamics simulations
Source: Mol Genet Genomic Med. 2019 Jul 25;7(9):e902. doi: 10.1002/mgg3.902 (PMC6732295; doi:10.1002/mgg3.902)

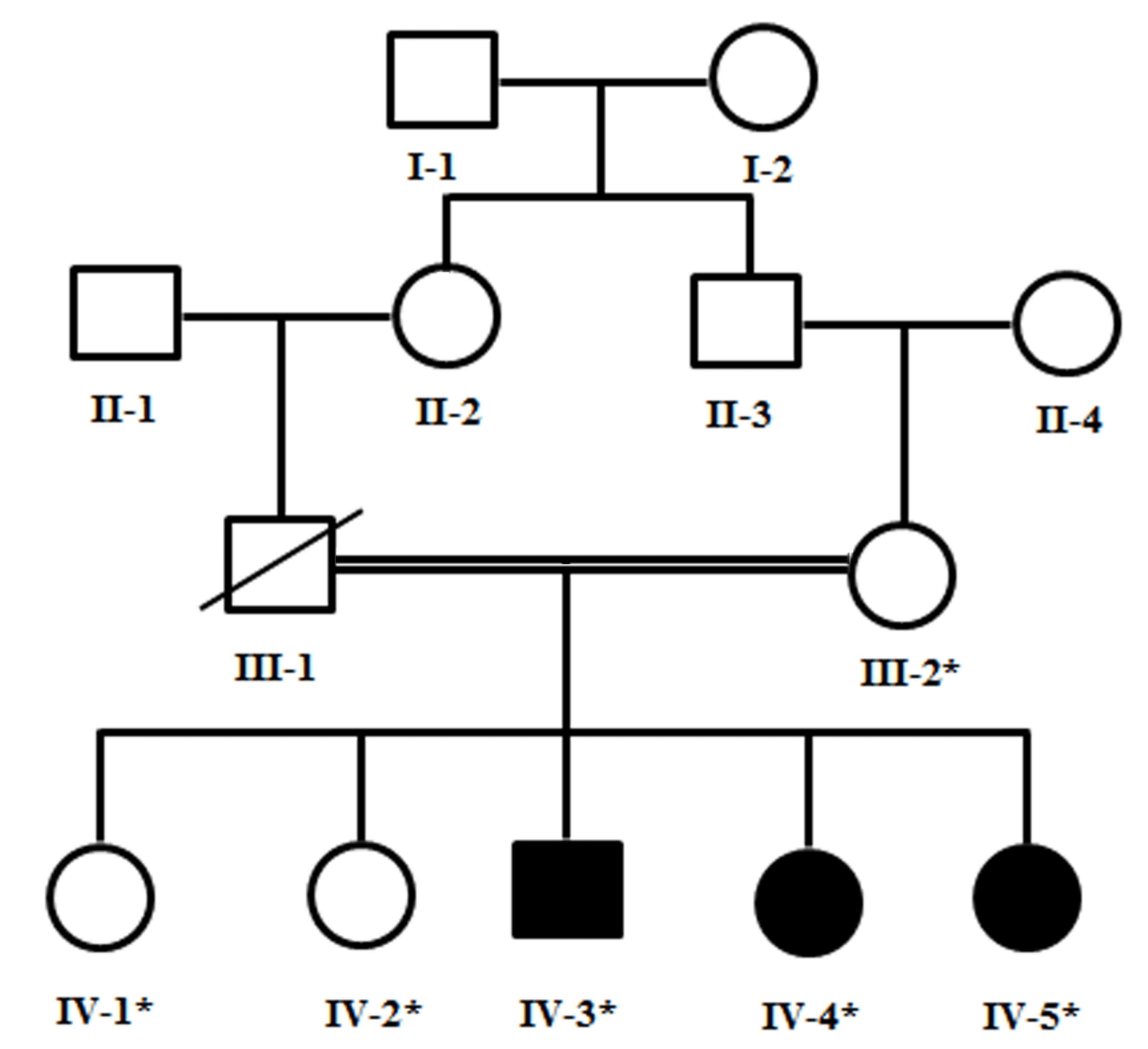

Supplement: Supplementary file 1 [file MGG3-7-e902-s001.png]

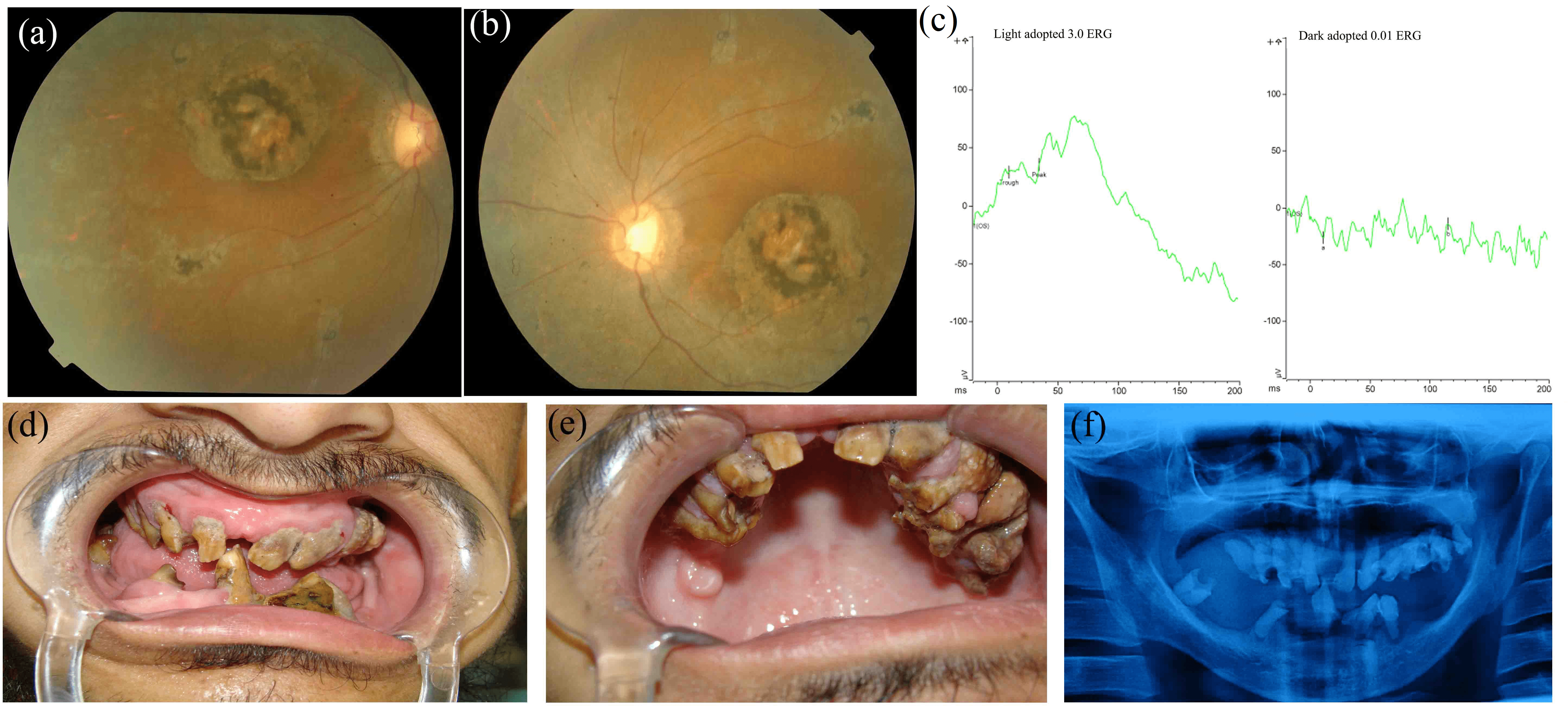

Supplement: Supplementary file 2 [file MGG3-7-e902-s002.png]

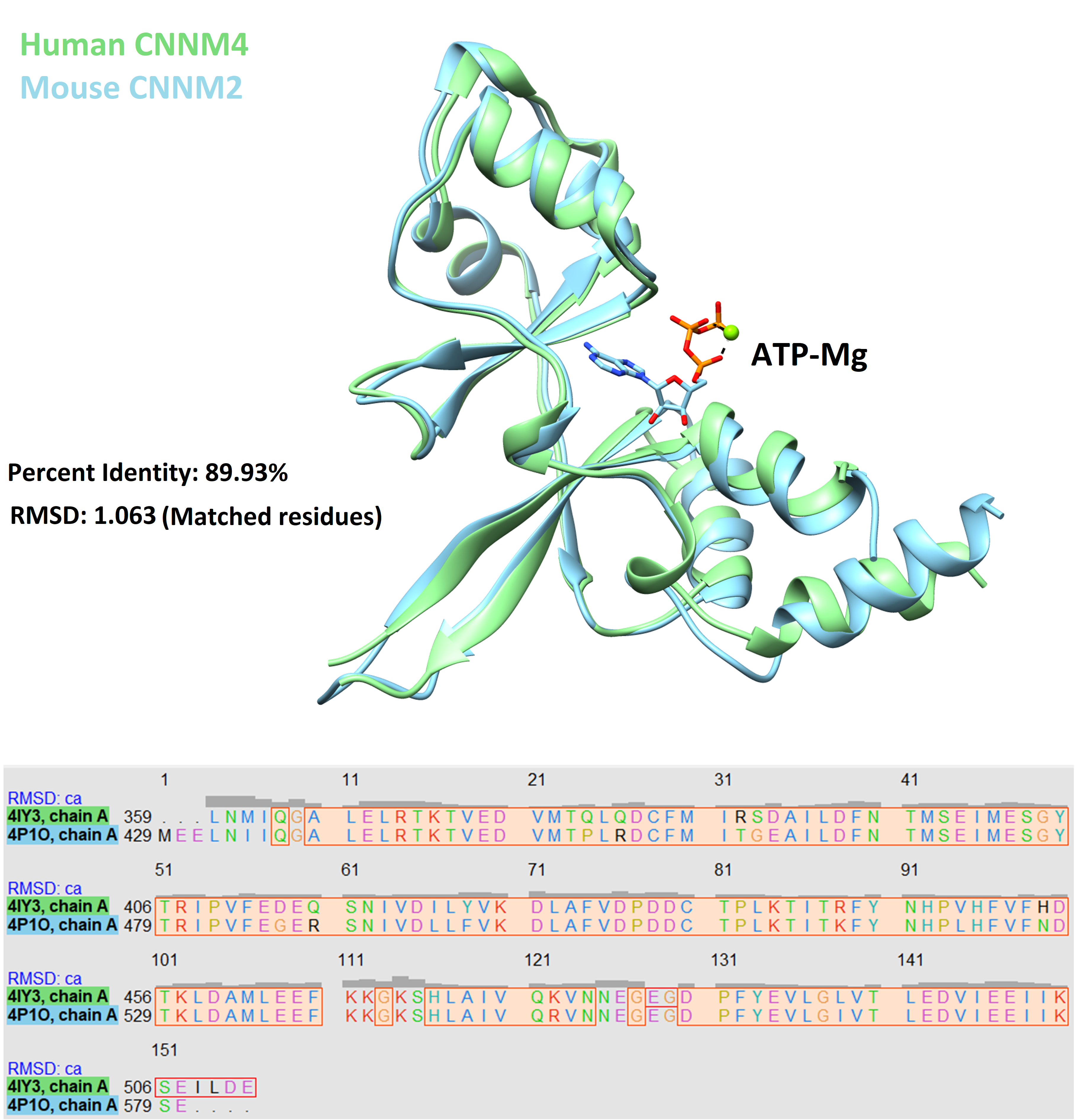

Supplement: Supplementary file 3 [file MGG3-7-e902-s003.png]

**A)**
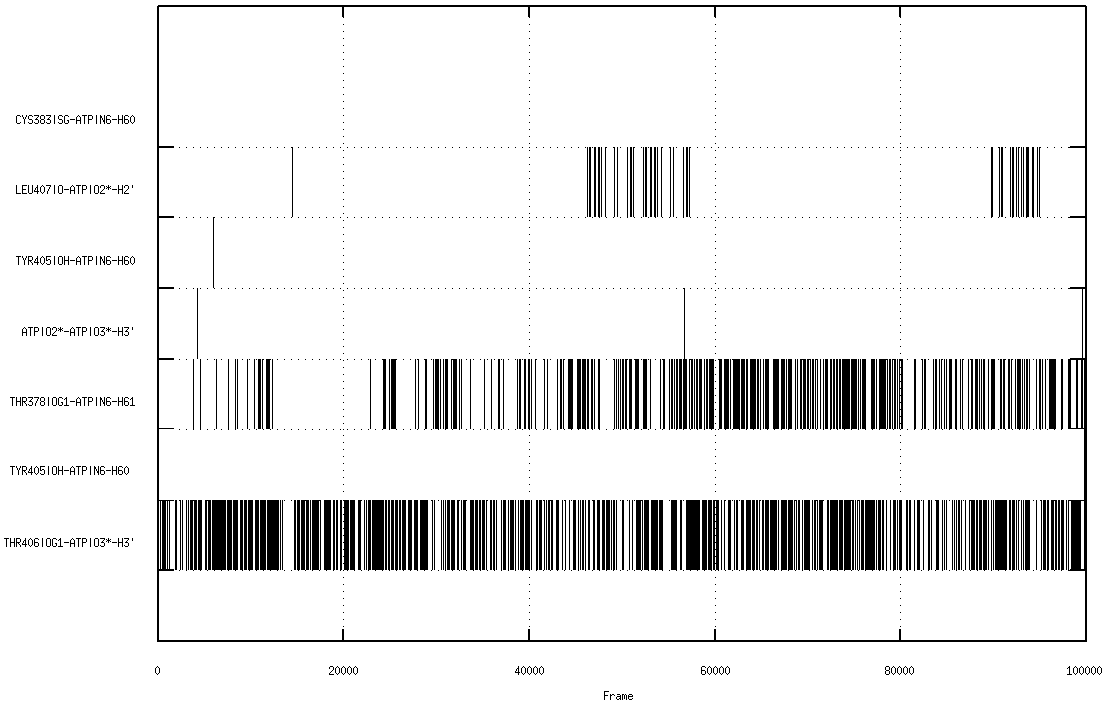


**B)**

**
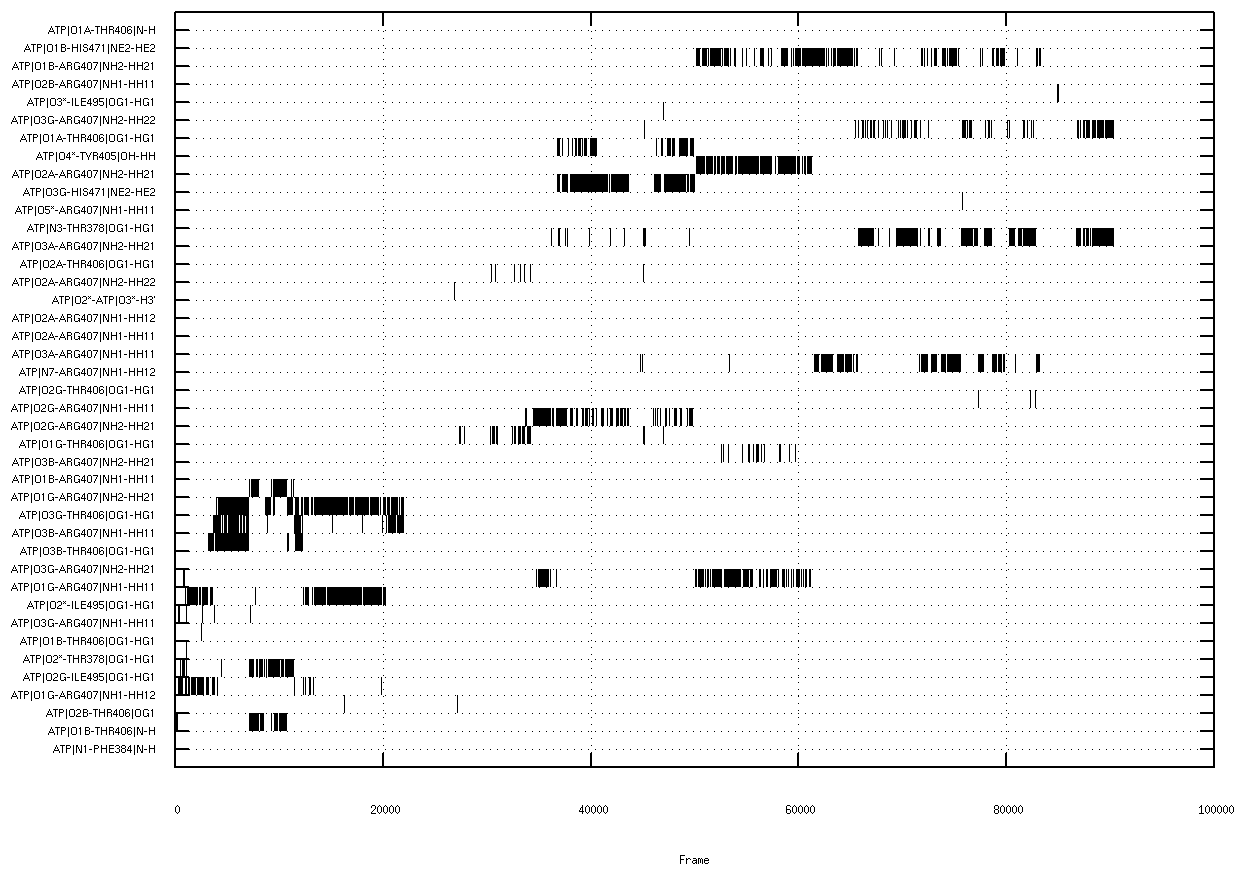
**

**C)
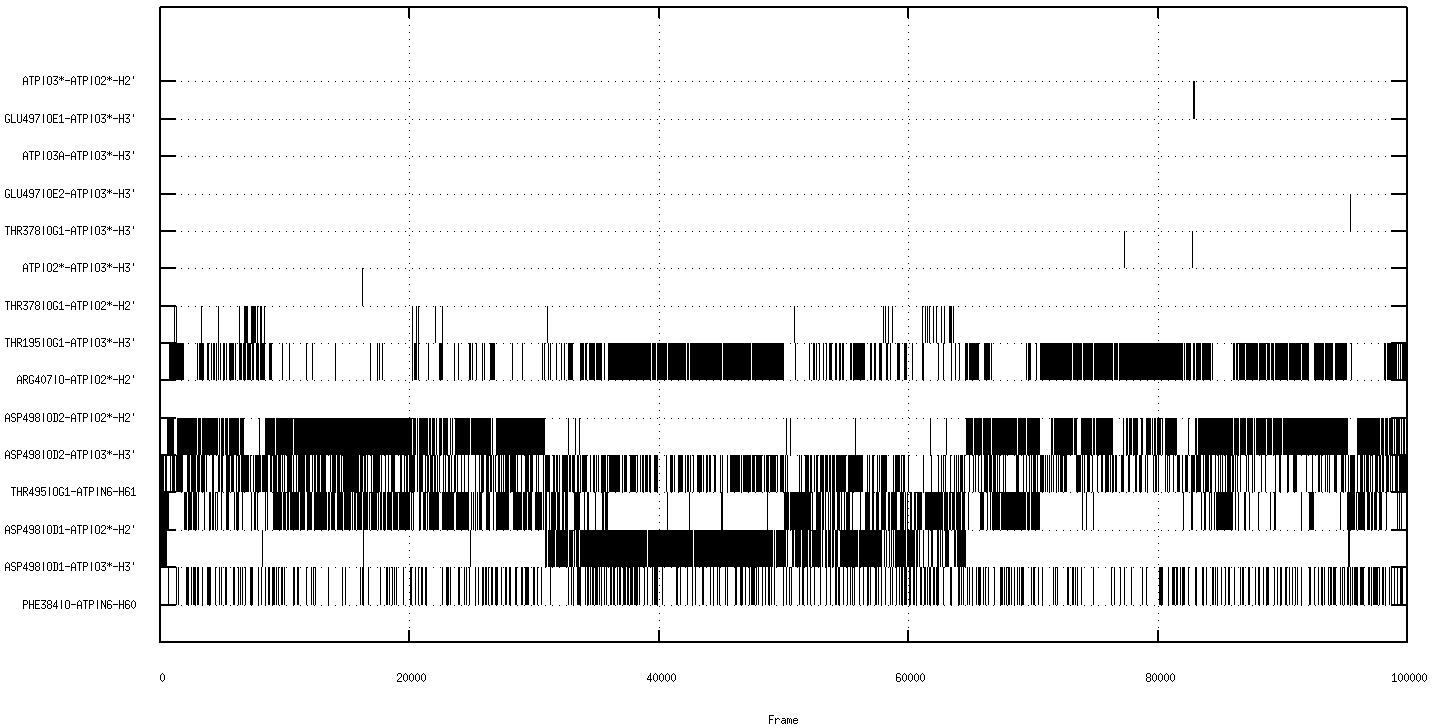
**

Supplement: Supplementary file 4 [file MGG3-7-e902-s004.docx]
